# Triaqua­(1,10-phenanthroline-2,9-dicarboxyl­ato)cobalt(II) dihydrate

**DOI:** 10.1107/S1600536810007567

**Published:** 2010-03-06

**Authors:** Zi-Fa Shi, Zhu-Qing Gao, Jin-Zhong Gu

**Affiliations:** a State Key Laboratory of Applied Organic Chemistry and College of Chemistry and Chemical Engineering, Lanzhou University, Lanzhou, Gansu 730000, People’s Republic of China; b School of Chemistry and Biology Engineering, Taiyuan University of Science and, Technology, Taiyuan 030021, People’s Republic of China

## Abstract

The title compound, [Co(C_14_H_6_N_2_O_4_)(H_2_O)_3_]·2H_2_O, has two­fold crystallographic symmetry. The Co^II^ atom is in a distorted penta­gonal-bipyramidal coordination environment with two N atoms and two O atoms from a tetradentate 1,10-phenanthroline-2,9-dicarboxyl­ate ligand and one O atom from a water mol­ecule forming the penta­gonal plane, and two O atoms from two water mol­ecules occupying axial positions. In the crystal, adjacent mol­ecules are linked by O—H⋯O hydrogen bonds, forming a three-dimensional network.

## Related literature

For the structures and properties of coordination compounds, see: Zhao *et al.* (2008 ▶); Poulsen *et al.* (2005 ▶). For the use of multi-carboxyl­ate and heterocyclic carboxylic acids in coordination chemistry, see: Luo *et al.* (2009 ▶); Han *et al.* (2009 ▶) and for the dicarboxyl­ate ligand H_2_PDA (H_2_PDA is 1,10-phenanthroline-2,9-dicarboxylic acid), see: Xie *et al.* (2005 ▶). For the isotypic structure [Mg(PDA)(H_2_O)_3_]·2H_2_O, see: Park *et al.* (2001 ▶). For the high affinity of the Co^II^ ion to water mol­ecules, see: (Zhang & Chen (2009 ▶). For bond distances and angles in other seven-coordinated Co^II^ complexes, see: Newkome *et al.* (1984 ▶); Rajput & Biradha (2007 ▶). For the synthesis of 1,10-phenanthroline-2,9-dicarboxylic acid, see: De Cian *et al.* (2007 ▶).
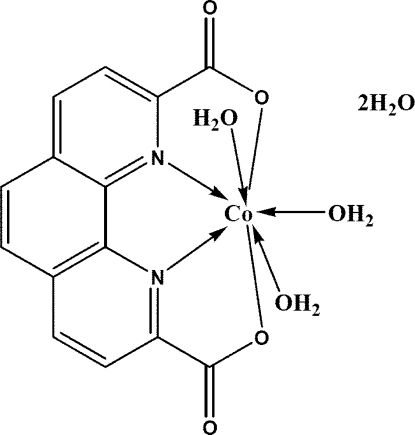


## Experimental

### 

#### Crystal data

[Co(C_14_H_6_N_2_O_4_)(H_2_O)_3_]·2H_2_O
*M* *_r_* = 415.22
Orthorhombic, 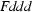

*a* = 7.4093 (5) Å
*b* = 18.9267 (17) Å
*c* = 46.609 (4) Å
*V* = 6536.1 (9) Å^3^
*Z* = 16
Mo *K*α radiation
μ = 1.11 mm^−1^
*T* = 296 K
0.20 × 0.19 × 0.17 mm

#### Data collection

Bruker SMART CCD diffractometer
9724 measured reflections
1877 independent reflections
1520 reflections with *I* > 2σ(*I*)
*R*_int_ = 0.032

#### Refinement

*R*[*F* ^2^ > 2σ(*F* ^2^)] = 0.035
*wR*(*F* ^2^) = 0.098
*S* = 1.06
1877 reflections
132 parameters
2 restraints
H atoms treated by a mixture of independent and constrained refinement
Δρ_max_ = 0.96 e Å^−3^
Δρ_min_ = −0.46 e Å^−3^

### 

Data collection: *SMART* (Bruker, 1997 ▶); cell refinement: *SAINT* (Bruker, 1997 ▶); data reduction: *SAINT*; program(s) used to solve structure: *SHELXS97* (Sheldrick, 2008 ▶); program(s) used to refine structure: *SHELXL97* (Sheldrick, 2008 ▶); molecular graphics: *SHELXTL* (Sheldrick, 2008 ▶); software used to prepare material for publication: *publCIF* (Westrip, 2010 ▶).

## Supplementary Material

Crystal structure: contains datablocks I, global. DOI: 10.1107/S1600536810007567/hg2639sup1.cif

Structure factors: contains datablocks I. DOI: 10.1107/S1600536810007567/hg2639Isup2.hkl

Additional supplementary materials:  crystallographic information; 3D view; checkCIF report

## Figures and Tables

**Table 1:** Hydrogen-bond geometry (Å, °)

*D*—H⋯*A*	*D*—H	H⋯*A*	*D*⋯*A*	*D*—H⋯*A*
O3—H3*A*⋯O1^i^	0.837 (17)	1.957 (16)	2.778 (2)	167 (2)
O5—H5*A*⋯O2^ii^	0.91	1.95	2.837 (3)	164
O5—H5*B*⋯O5^iii^	0.91	1.93	2.803 (4)	161
O5—H5*B*′⋯O5^ii^	0.90	2.22	3.096 (6)	165
O4—H4*A*⋯O2^iv^	0.74 (4)	2.02 (4)	2.750 (3)	171 (4)
O4—H4*B*⋯O5	0.79 (4)	2.04 (4)	2.818 (3)	169 (3)

Symmetry codes: (i) 
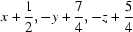
; (ii) 
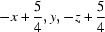
; (iii) 
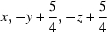
; (iv) 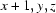.

**Fig. 1.**
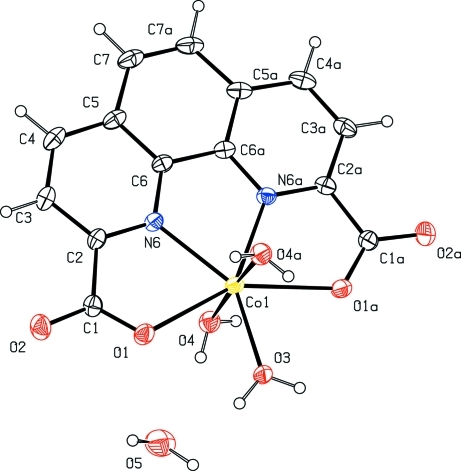
The molecular structure of (I) showing 50% proability displacement ellipsoids and the atom-numbering scheme (Symmetry code A: -x+7/4,-y+7/4,z).

## supplementary crystallographic information

### Comment

In recent years, the research of coordination compounds has been one of the most attractive fields due to their peculiar structures and properties (Zhao *et al.*, 2008; Poulsen *et al.*, 2005). Many multi-carboxylate or heterocyclic carboxylic acids are used for this purpose (Luo *et al.*, 2009; Han *et al.*, 2009). In the designed synthesis of the coordination compounds, H_2_PDA is an excellent dicarboxylate ligand (Xie *et al.*, 2005). In order to extend the investigation, we have prepared the Co^II^ complex of H_2_PDA, and report its crystal structure here.

The title compound (Fig. 1) is located on a twofold axis of symmetry which passes through the Co and O3 atoms, which is isomorphous with [Mg(PDA)(H_2_O)_3_].2H_2_O (Park *et al.*, 2001). The seven-coordinated Co atom is in a distorted pentagonal bipyramidal geometry. Two N and two O atoms from PDA and one O atom from a water molecule define the pentagonal plane, and the two axial positions are occupied by O atoms derived from two water molecules.

Important bond distances and angles are presented in Table 1. The bond distances between Co and the PDA donor atoms [Co—O1 2.3364 (16) Å and Co—N6 2.1936 (18) Å] are significantly longer than those to the coordinated water molecules [Co—O3 2.072 (2) Å and Co—O4 2.1254 (19) Å]. This is probably due to the high rigidity of PDA as well as the high affinity of the Co^II^ ion to water molecules (Zhang & Chen, 2009). The carboxylate groups of the PDA ligand are almost coplanar with the phenanthroline unit as indicated by the O1—C1—C2—N6 torsion angle of 2.6 (3)°. All bond distances and angles are similar to those observed in other seven-coordinated Co^II^ complexes (Newkome *et al.*, 1984; Rajput & Biradha, 2007). Adjacent molecules are linked by O—H···O hydrogen bonds, forming a three-dimensional network.

### Experimental

1,10-Phenanthroline-2,9-dicarboxylic acid was synthesized by using a literature method (De Cian *et al.*, 2007). To a solution of cobalt nitrate hexahydrate (0.145 g, 0.5 mmol) in water (5 ml) was added an aqueous solution (5 ml) of the ligand (0.135 g, 0.5 mmol) and sodium hydroxide (0.04 g, 1.0 mmol). The reactants were sealed in a 25-ml Teflon-lined, stainless-steel Parr bomb. The bomb was heated at 433 K for 3 days. The cool solution yielded single crystals in ca 50% yield. Anal. Calcd for C_14_H_16_CoN_2O_9: C, 40.50; H, 3.88; N, 6.75. Found: C, 40.01; H, 4.12; N, 6.45.

### Refinement

The coordinated water H atoms were located in a difference Fourier map and refined with distance constraints of O—H = 0.83 (3) Å. The free water H atoms attached to oxygen atoms were placed at calculated positions and refined with the riding model, considering the position of oxygen atoms and the quantity of H atoms. The carbon-bound H atoms were placed in geometrically idealized positions, with C—H = 0.93 Å, and constrained to ride on their respective parent atoms, with *U*iso(H) = 1.2 *U*eq(C).

### Crystal data

**Table:**

[Co(C_14_H_6_N_2_O_4_)(H_2_O)_3_]·2H_2_O	*F*(000) = 3408
*M**_r_* = 415.22	*D*_x_ = 1.688 Mg m^−^^3^
Orthorhombic, *F**d**d**d*	Mo *K*α radiation, λ = 0.71073 Å
Hall symbol: -F 2uv 2vw	Cell parameters from 2624 reflections
*a* = 7.4093 (5) Å	θ = 3.0–25.2°
*b* = 18.9267 (17) Å	µ = 1.11 mm^−^^1^
*c* = 46.609 (4) Å	*T* = 296 K
*V* = 6536.1 (9) Å^3^	Block, yellow
*Z* = 16	0.20 × 0.19 × 0.17 mm

### Data collection

**Table:**

Bruker APEXII CCD diffractometer	1520 reflections with *I* > 2σ(*I*)
Radiation source: fine-focus sealed tube	*R*_int_ = 0.032
graphite	θ_max_ = 27.5°, θ_min_ = 1.8°
φ and ω scans	*h* = −9→9
9724 measured reflections	*k* = −24→23
1877 independent reflections	*l* = −45→60

### Refinement

**Table:**

Refinement on *F*^2^	Primary atom site location: structure-invariant direct methods
Least-squares matrix: full	Secondary atom site location: difference Fourier map
*R*[*F*^2^ > 2σ(*F*^2^)] = 0.035	Hydrogen site location: inferred from neighbouring sites
*wR*(*F*^2^) = 0.098	H atoms treated by a mixture of independent and constrained refinement
*S* = 1.06	*w* = 1/[σ^2^(*F*_o_^2^) + (0.0494*P*)^2^ + 16.2882*P*] where *P* = (*F*_o_^2^ + 2*F*_c_^2^)/3
1877 reflections	(Δ/σ)_max_ = 0.001
132 parameters	Δρ_max_ = 0.96 e Å^−^^3^
2 restraints	Δρ_min_ = −0.46 e Å^−^^3^

### Special details

**Table:**

Geometry. All esds (except the esd in the dihedral angle between two l.s. planes) are estimated using the full covariance matrix. The cell esds are taken into account individually in the estimation of esds in distances, angles and torsion angles; correlations between esds in cell parameters are only used when they are defined by crystal symmetry. An approximate (isotropic) treatment of cell esds is used for estimating esds involving l.s. planes.
Refinement. Refinement of *F*^2^ against ALL reflections. The weighted *R*-factor wR and goodness of fit *S* are based on *F*^2^, conventional *R*-factors *R* are based on *F*, with *F* set to zero for negative *F*^2^. The threshold expression of *F*^2^ > σ(*F*^2^) is used only for calculating *R*-factors(gt) etc. and is not relevant to the choice of reflections for refinement. *R*-factors based on *F*^2^ are statistically about twice as large as those based on *F*, and *R*- factors based on ALL data will be even larger.

### Fractional atomic coordinates and isotropic or equivalent isotropic displacement parameters (Å^2^)

**Table:**

	*x*	*y*	*z*	*U*_iso_*/*U*_eq_	Occ. (<1)
Co1	0.8750	0.8750	0.575281 (8)	0.02703 (15)	
C1	0.5006 (3)	0.79758 (12)	0.56914 (5)	0.0303 (5)	
C2	0.5619 (3)	0.81188 (11)	0.53888 (5)	0.0286 (5)	
C3	0.4668 (3)	0.79146 (13)	0.51428 (5)	0.0369 (5)	
H3	0.3573	0.7678	0.5160	0.044*	
C4	0.5356 (4)	0.80639 (13)	0.48763 (5)	0.0403 (6)	
H4	0.4723	0.7936	0.4712	0.048*	
C5	0.7025 (3)	0.84111 (12)	0.48536 (5)	0.0336 (5)	
C6	0.7884 (3)	0.85853 (11)	0.51113 (4)	0.0283 (5)	
C7	0.7938 (4)	0.85890 (13)	0.45911 (5)	0.0426 (6)	
H7	0.7394	0.8479	0.4417	0.051*	
H3A	0.953 (3)	0.8937 (8)	0.6302 (4)	0.063 (10)*	
H5A	0.8692	0.7242	0.6386	0.075*	
H5B	0.8592	0.6518	0.6227	0.075*	0.50
H5B'	0.7138	0.7061	0.6221	0.075*	0.50
H4A	1.088 (5)	0.7728 (17)	0.5770 (7)	0.049 (10)*	
H4B	0.955 (5)	0.7546 (18)	0.5909 (8)	0.060 (11)*	
N6	0.7184 (2)	0.84503 (10)	0.53724 (4)	0.0274 (4)	
O1	0.6029 (2)	0.82142 (9)	0.58824 (4)	0.0380 (4)	
O2	0.3589 (2)	0.76361 (11)	0.57280 (4)	0.0446 (5)	
O3	0.8750	0.8750	0.61974 (5)	0.0459 (7)	
O4	0.9892 (3)	0.77204 (10)	0.57658 (4)	0.0359 (4)	
O5	0.8336 (4)	0.69890 (11)	0.62306 (5)	0.0670 (7)	

### Atomic displacement parameters (Å^2^)

**Table:**

	*U*^11^	*U*^22^	*U*^33^	*U*^12^	*U*^13^	*U*^23^
Co1	0.0278 (3)	0.0337 (3)	0.0196 (2)	−0.00067 (18)	0.000	0.000
C1	0.0231 (11)	0.0333 (12)	0.0344 (12)	0.0020 (9)	0.0013 (9)	−0.0046 (9)
C2	0.0256 (11)	0.0298 (11)	0.0305 (11)	0.0043 (9)	−0.0041 (9)	−0.0060 (8)
C3	0.0325 (13)	0.0401 (13)	0.0382 (13)	−0.0006 (10)	−0.0095 (10)	−0.0084 (10)
C4	0.0463 (15)	0.0418 (14)	0.0328 (13)	0.0055 (11)	−0.0153 (11)	−0.0087 (10)
C5	0.0438 (14)	0.0321 (12)	0.0248 (11)	0.0101 (11)	−0.0069 (9)	−0.0048 (9)
C6	0.0313 (12)	0.0296 (11)	0.0239 (10)	0.0057 (9)	−0.0010 (9)	−0.0015 (8)
C7	0.0637 (17)	0.0412 (14)	0.0228 (11)	0.0069 (12)	−0.0065 (11)	−0.0037 (9)
N6	0.0261 (9)	0.0320 (9)	0.0241 (9)	0.0013 (8)	−0.0014 (7)	−0.0023 (7)
O1	0.0327 (9)	0.0544 (10)	0.0268 (8)	−0.0075 (8)	0.0024 (7)	−0.0060 (7)
O2	0.0304 (10)	0.0550 (11)	0.0483 (11)	−0.0086 (8)	0.0052 (8)	−0.0053 (8)
O3	0.0490 (16)	0.0680 (17)	0.0206 (11)	−0.0285 (14)	0.000	0.000
O4	0.0312 (11)	0.0403 (10)	0.0363 (10)	0.0012 (8)	−0.0012 (8)	0.0004 (8)
O5	0.1009 (19)	0.0418 (11)	0.0582 (13)	−0.0027 (12)	0.0008 (13)	−0.0004 (10)

### Geometric parameters (Å, °)

**Table:**

Co1—O3	2.072 (2)	C4—C5	1.404 (4)
Co1—O4	2.1254 (19)	C4—H4	0.9300
Co1—O4^i^	2.1254 (19)	C5—C6	1.399 (3)
Co1—N6^i^	2.1936 (18)	C5—C7	1.438 (3)
Co1—N6	2.1936 (18)	C6—N6	1.348 (3)
Co1—O1	2.3364 (16)	C6—C6^i^	1.426 (5)
Co1—O1^i^	2.3364 (16)	C7—C7^i^	1.349 (6)
C1—O2	1.243 (3)	C7—H7	0.9300
C1—O1	1.253 (3)	O3—H3A	0.837 (17)
C1—C2	1.506 (3)	O4—H4A	0.74 (4)
C2—N6	1.320 (3)	O4—H4B	0.79 (4)
C2—C3	1.400 (3)	O5—H5A	0.9066
C3—C4	1.372 (4)	O5—H5B	0.9119
C3—H3	0.9300	O5—H5B'	0.8988
			
O3—Co1—O4	88.37 (5)	C4—C3—C2	119.8 (2)
O3—Co1—O4^i^	88.37 (5)	C4—C3—H3	120.1
O4—Co1—O4^i^	176.75 (11)	C2—C3—H3	120.1
O3—Co1—N6^i^	143.92 (5)	C3—C4—C5	119.5 (2)
O4—Co1—N6^i^	92.83 (8)	C3—C4—H4	120.3
O4^i^—Co1—N6^i^	89.80 (7)	C5—C4—H4	120.3
O3—Co1—N6	143.92 (5)	C6—C5—C4	116.5 (2)
O4—Co1—N6	89.80 (7)	C6—C5—C7	117.5 (2)
O4^i^—Co1—N6	92.83 (8)	C4—C5—C7	126.0 (2)
N6^i^—Co1—N6	72.16 (10)	N6—C6—C5	123.7 (2)
O3—Co1—O1	75.02 (4)	N6—C6—C6^i^	115.43 (12)
O4—Co1—O1	86.46 (8)	C5—C6—C6^i^	120.83 (14)
O4^i^—Co1—O1	92.69 (7)	C7^i^—C7—C5	121.69 (15)
N6^i^—Co1—O1	141.06 (6)	C7^i^—C7—H7	119.2
N6—Co1—O1	68.91 (6)	C5—C7—H7	119.2
O3—Co1—O1^i^	75.02 (4)	C2—N6—C6	118.72 (18)
O4—Co1—O1^i^	92.69 (7)	C2—N6—Co1	122.73 (14)
O4^i^—Co1—O1^i^	86.46 (8)	C6—N6—Co1	118.50 (15)
N6^i^—Co1—O1^i^	68.91 (6)	C1—O1—Co1	119.62 (15)
N6—Co1—O1^i^	141.06 (6)	Co1—O3—H3A	125.6 (15)
O1—Co1—O1^i^	150.03 (8)	Co1—O4—H4A	112 (3)
O2—C1—O1	126.8 (2)	Co1—O4—H4B	106 (2)
O2—C1—C2	118.4 (2)	H4A—O4—H4B	108 (3)
O1—C1—C2	114.7 (2)	H5A—O5—H5B	118.1
N6—C2—C3	121.7 (2)	H5A—O5—H5B'	104.2
N6—C2—C1	113.83 (18)	H5B—O5—H5B'	110.7
C3—C2—C1	124.5 (2)		
			
O2—C1—C2—N6	−176.6 (2)	C6^i^—C6—N6—Co1	0.2 (3)
O1—C1—C2—N6	2.6 (3)	O3—Co1—N6—C2	−2.8 (2)
O2—C1—C2—C3	2.2 (3)	O4—Co1—N6—C2	84.14 (17)
O1—C1—C2—C3	−178.6 (2)	O4^i^—Co1—N6—C2	−93.94 (17)
N6—C2—C3—C4	−0.5 (4)	N6^i^—Co1—N6—C2	177.2 (2)
C1—C2—C3—C4	−179.2 (2)	O1—Co1—N6—C2	−2.14 (16)
C2—C3—C4—C5	1.0 (4)	O1^i^—Co1—N6—C2	178.19 (14)
C3—C4—C5—C6	−0.1 (3)	O3—Co1—N6—C6	179.93 (11)
C3—C4—C5—C7	178.5 (2)	O4—Co1—N6—C6	−93.11 (16)
C4—C5—C6—N6	−1.3 (3)	O4^i^—Co1—N6—C6	88.81 (16)
C7—C5—C6—N6	179.9 (2)	N6^i^—Co1—N6—C6	−0.07 (11)
C4—C5—C6—C6^i^	177.6 (2)	O1—Co1—N6—C6	−179.40 (17)
C7—C5—C6—C6^i^	−1.2 (4)	O1^i^—Co1—N6—C6	0.9 (2)
C6—C5—C7—C7^i^	0.1 (4)	O2—C1—O1—Co1	174.59 (19)
C4—C5—C7—C7^i^	−178.6 (3)	C2—C1—O1—Co1	−4.5 (3)
C3—C2—N6—C6	−0.9 (3)	O3—Co1—O1—C1	−176.70 (18)
C1—C2—N6—C6	178.00 (18)	O4—Co1—O1—C1	−87.44 (18)
C3—C2—N6—Co1	−178.11 (16)	O4^i^—Co1—O1—C1	95.71 (18)
C1—C2—N6—Co1	0.7 (3)	N6^i^—Co1—O1—C1	2.7 (2)
C5—C6—N6—C2	1.8 (3)	N6—Co1—O1—C1	3.71 (16)
C6^i^—C6—N6—C2	−177.2 (2)	O1^i^—Co1—O1—C1	−176.70 (18)
C5—C6—N6—Co1	179.17 (16)		

Symmetry codes: (i) −*x*+7/4, −*y*+7/4, *z*.

### Hydrogen-bond geometry (Å, °)

**Table:**

*D*—H···*A*	*D*—H	H···*A*	*D*···*A*	*D*—H···*A*
O3—H3A···O1^ii^	0.837 (17)	1.957 (16)	2.778 (2)	167 (2)
O5—H5A···O2^iii^	0.91	1.95	2.837 (3)	164
O5—H5B···O5^iv^	0.91	1.93	2.803 (4)	161
O5—H5B'···O5^iii^	0.90	2.22	3.096 (6)	165
O4—H4A···O2^v^	0.74 (4)	2.02 (4)	2.750 (3)	171 (4)
O4—H4B···O5	0.79 (4)	2.04 (4)	2.818 (3)	169 (3)

Symmetry codes: (ii) *x*+1/2, −*y*+7/4, −*z*+5/4; (iii) −*x*+5/4, *y*, −*z*+5/4; (iv) *x*, −*y*+5/4, −*z*+5/4; (v) *x*+1, *y*, *z*.
